# Importance of *ROS1* gene fusions in non-small cell lung cancer

**DOI:** 10.20517/cdr.2022.105

**Published:** 2023-06-09

**Authors:** Meri Muminovic, Carlos Rodrigo Carracedo Uribe, Andres Alvarez-Pinzon, Khine Shan, Luis E. Raez

**Affiliations:** ^1^Department of Hematology-Oncology, Memorial Cancer Institute/Memorial Health Care System, Florida International University, Pembroke Pines, FL 33028, USA.; ^2^Department of Internal Medicine, Memorial Health Care System, Florida International University, Pembroke Pines, FL 33028, USA.; ^3^Office of Human Research, Memorial Healthcare System, Pembroke Pines, FL 33028, USA.

**Keywords:** *ROS1*, targeted therapy, non-small cell lung cancer

## Abstract

Targeted therapy has become one of the standards of care for advanced lung cancer. More than 10 genetic aberrations have been discovered that are actionable and several tyrosine kinase inhibitors (TKIs) have been approved to target each of them. Among several genetic aberrations that are actionable in non-small cell lung cancer (NSCLC), *ROS1* translocations also known as gene fusion proteins, are found in only 1%-2% of the patient population. *ROS1* mutations can usually be detected using a combination of techniques such as immunohistochemistry (IHC), Fluorescence in-situ testing (FISH), polymerase chain reaction (PCR), and next-generation sequencing (NGS). However, RNA NGS and ctDNA NGS (liquid biopsies) also contribute to the diagnosis. There are currently numerous FDA-approved agents for these tumors, including crizotinib and entrectinib; however, there is in-vitro sensitivity data and clinical data documenting responses to ceritinib and lorlatinib. Clinical responses and survival rates with these agents are frequently among the best compared to other TKIs with genetic aberrations; however, intrinsic or extrinsic mechanisms of resistance may develop, necessitating research for alternative treatment modalities. To combat the mechanisms of resistance, novel agents such as repotrectenib, cabozantinib, talotrectinib, and others are being developed. In this article, we examine the literature pertaining to patients with *ROS1* tumors, including epidemiology, clinical outcomes, resistance mechanisms, and treatment options.

## INTRODUCTION

Cancer is one of the leading causes of death in the United States. Over 1.8 million people are newly diagnosed with cancer every year, and 606,520 people lose their lives as a direct result of the disease. Three most common types of cancer are breast cancer, lung cancer, and prostate cancer^[1]^. There were 236,740 new cases of lung cancer, and over 130,180 people lost their lives to the disease^[2]^. Lung cancer is the most common cause of death resulting from any form of cancer, including breast, colorectal, prostate, and brain cancers.

Cancer of the lung is a disease that originates in the pulmonary parenchyma or the airways of the lungs. Non-small cell lung cancer (NSCLC) and small cell lung cancer (SCLC), which together account for 95% of all cases of lung cancer, are the two types of lung cancer that can be classified based on the histology. NSCLC includes adenocarcinoma, squamous cell carcinoma, adenosquamous carcinoma, large cell carcinoma and sarcomatoid.

Previously, the treatment for lung cancer was only systemic chemotherapy that would target all cells that were proliferating or dividing which would prolong survival but would cause burden on quality of life of patients due to the toxic effects of treatment. Most recently, there has been a substantial amount of research on molecular pathways which allowed for target therapies to be developed to combat the pathways. Common molecular targets in lung cancer include EGFR, ALK, KRAS, ROS1, BRAF V600E, NTRK 1/2/3, METexon14, RET, ERBB2 and PD-L1. Molecular testing can be done with DNA sequencing, next generation sequencing (NGS), Fluorescence in-situ testing (FISH), immunohistochemistry (IHC) and liquid biopsies.

The availability of molecular testing, the identification of mutational drivers, and the introduction of targeted therapies have all changed the landscape of lung cancer therapy in recent years^[1]^. Epidermal Growth Factor Receptor (EGFR) mutations, for example, are known to contribute to the maintenance and functioning of cancer stem cells, including metabolism, immunomodulatory activity, dormancy, and therapeutic resistance^[1]^. While the *ALK* gene, located on the short arm of chromosome 2, is a transmembrane tyrosine kinase receptor which activates downstream signaling pathways resulting in uncontrolled cell proliferation and survival have been studied previously and are good therapeutic targets in the treatment of NSCLC^[3,4]^. The effectiveness of targeted therapy in treating EGFR^[2-6]^ and ALK^[7-11]^ mutations has increased interest in identifying additional oncogenic drivers with potential for clinical use.

The proto-oncogene *ROS1* on chromosome 6 encodes a tyrosine kinase receptor, limited to distinct epithelial cells during embryonic development^[5]^ but with unknown functionality afterwards. It was originally identified in 1986 as the cellular homolog of the transforming *v-ro*s sequence from the UR2 avian sarcoma virus^[6]^ and later identified in glioblastoma-derived cells^[7]^. Further research has demonstrated ROS1’s ability to cause cancer using in vitro and in vivo animal models^[12]^. The discovery of *ROS1* gene fusions in NSCLC has important clinical implications because it allows for the treatment of a subset of patients with a targeted therapy that can significantly improve their outcomes. It emphasizes the significance of extensive molecular profiling in NSCLC for identifying targetable mutations and guiding treatment decisions. Additionally, TKIs are effective on multiple targets including targeting the ALK rearrangement position, ROS1 fusions and NTRK rearrangements using agents such as ceritinib, crizotinib, lorlatinib and entrectinib^[8]^.

Therapy options for NSCLC includes chemotherapy, immunotherapy and targeted therapy. Common chemotherapy options includes platinum agents (cisplatin, carboplatin) in combinations with taxanes (paclitaxel, albumin bound paclitaxel) or pemetrexed. Immunotherapy may be incorporated also using pembrolizumab, atezolizumab or even ipilimumab and nivolumab.

### Molecular testing

Molecular testing is constantly evolving field and there is no single standard modality for detection of abnormalities. Different modalities of testing include DNA sequencing, NGS, FISH, IHC and liquid biopsies. DNA sequencing is the one of the oldest forms of testing for mutations which looks at an entire length of a single gene for mutation. The sensitivity is the lowest amongst all tests and may cause false negatives. The test requires the tumor cellularity to be high in the tissue sample to detect an abnormality. DNA and RNA NGS allows for testing of multiple genes at the same time or whole genomes with high sensitivity even if the tumor cellularity is low. The sensitivity is so high that it may detect even molecular alterations in the blood via circulating tumor DNA. FISH helps to examine gene rearrangements such as translocations, amplifications or deletions using DNA probes of various colors that move apart when gene has separated. IHC is both sensitive and specific. The turnaround time for results is rapid and it is the only test available to test for PD-L1 expression. Lastly, liquid biopsies allow for non-invasive and inexpensive means to test even when there is minimal tumor samples available for testing. It allows to monitor for disease response during treatment or even relapse in the future^[9]^. Liquid biopsies allow for detection of cell-free ctDNA in the blood of lung cancer patients. The negative aspects of the test is that there is a high false negative rate compared to standard tests due to small and variable amounts of circulating DNA that may be present. The sensitivity of liquid biopsies is 60% to 80% and sometimes cannot detect tumors that do not secrete DNA into the blood.

## NSCLC GENOTYPES

### ROS1 gene fusions

*ROS1* gene fusions are genetic alterations that have been associated with the development and progression of multiple cancers, including NSCLC. The *ROS1* gene located on chromosome 6 (region 6q22.1) is responsible for the generation of two main splice variants of ROS1 that are encoded by either exon 43 or exon 44 and codes for a receptor tyrosine kinase that is essential for cell growth and differentiation. *ROS1* gene fusions occur when a portion of the gene joins with another gene, resulting in the production of a new, chimeric protein that promotes the growth of cancerous cells. *ROS1* fusion genes have been linked to a wide range of cancers ever since their discovery in the glioblastoma cell line U118MG in 1987^[10-12]^. *ROS1* is a proto-oncogene that encodes a receptor tyrosine kinase in humans, but its physiological function is not known. The somatic chromosomal fusions that involve ROS1 produce chimeric oncoproteins that are the driving force behind a wide variety of cancers, and a significant amount of interpatient partner-gene heterogeneity has been observed in various types of cancer. While ROS1 rearrangements are only present in approximately 1%-2% of patients with lung cancer, a significant number of people are affected by this mutation given high prevalence of lung cancer.

ROS1 fusions are most common in patients with NSCLC who are younger (median age of 50 years) and who have never smoked (80%)^[12-15]^. The CD74-ROS1 fusion is the most prevalent type of ROS1 fusion (44%), followed by the EZR-ROS1 fusion (16%), SDC4-ROS1 fusion (14%), and SLC34A2-ROS1 fusion (10%). Interchromosomal translocations are the major cause of recurrent ROS1 fusions in NSCLCs^[12,15]^. In model systems, ROS1 fusions on their own are sufficient to induce tumorigenesis; however, *ROS1* fusions working in conjunction with other aberrant oncogenes or tumor suppressor pathways can promote a significantly more aggressive form of disease. In some instances, *ROS1* fusions have been discovered to coincide with the presence of other oncogenic alterations. Other driver mutations, such as RET, NTRK, and ALK fusions in NSCLCs, IMTs, and Spitzoid neoplasms, as well as FGFR2 and IDH alterations in cholangiocarcinoma^[13,14]^, are likely to be mutually exclusive with the ROS1 fusions. In clinical practice, ROS1 fusion can be evaluated on a tissue biopsy and fluid cytology with ROS1 IHC. However, similar to the ALK IHC, the ROS1 IHC can report false positive results and thus, requires confirmation with NGS, ROS1 FISH, or with a multiplex reverse transcriptase-polymerase chain reaction (RT-PCR) panel.

*ROS1* gene fusions in NSCLC are typically detected via molecular testing, such as FISH or RT-PCR. These techniques enable the identification of the precise fusion partner and the development of therapies that inhibit the activity of the chimeric protein and plasma samples, the NGS can be used to analyze ROS1 rearrangement^[13,14,16]^. *ROS1* gene fusions in NSCLC have a significant potential therapeutic application. ROS1 inhibitors have shown remarkable efficacy in patients with ROS1-positive NSCLC, with response rates ranging from 60% to 80% and a median progression-free survival (PFS) of 12-19 months. This suggests that ROS1 inhibitors are highly effective. This is a significant improvement over conventional chemotherapy, which only has a moderate impact on NSCLC. Certain minority groups, including African-Americans, are disproportionately affected by lung cancer and may be more likely to have *ROS1* gene fusions. Understanding the epidemiology of ROS1 mutations in lung cancer, particularly in minority populations, is critical for improving cancer prevention, diagnosis, and treatment outcomes; similarly, understanding the prevalence, types, and characteristics of ROS1 fusion-positive mutations in NSCLC is critical for developing effective, patient-beneficial targeted therapies^[13,16]^. The use of appropriate diagnostic techniques to identify ROS1 fusion-positive patients, as well as the use of ROS1 inhibitors, has shown tremendous promise in the treatment of NSCLC.

### EGFR mutations

Epidermal growth factor receptor (EGFR) mutations compromise 15% of NSCLC adenocarcinomas and occur often in non-smokers and Asian populations^[17]^. Common mutations in EGFR include exon 19 deletions or exon 21 L858R mutations which are very responsive to TKIs for treatment. Previously, first-generation TKI (gefitinib, erlotinib) and second-generation (afatinib) TKIs have been used for treatment but recently there has been improvement in survival with third-generation agents such as Osimertinib. Osimertinib is approved for first-line treatment in patients with EGFR exon 19 deletions or exon 21 L858R mutations. In the FLAURA study, a phase III trial, patients with treatment naïve EGFR-mutated NSCLC were randomized to receive Osimertinib versus the standard of care TKI (gefitnib or erlotinib). Osimertinib showed improvement in PFS 18.9 months versus 10.2 months, increased duration of response (DOR) (17.2 months versus 8.5 months) and overall survival (OS) 38.6 months versus 31.8 months^[18]^. Common adverse reactions include QTc prolongation and decreased left ventricular ejection fraction.

### ALK mutations

Anaplastic lymphoma kinase (ALK) is a tyrosine kinase that is often found to have chromosomal rearrangements involving *ALK* gene on chromosome 2 and compromise 3%-5% of NSCLCs^[19,20]^. The rearrangement involves the 5’ end of the echinoderm microtubule-associated protein-like (*EML4*) gene with the 3’ end of the *ALK* gene, creating a fusion oncogene *EML4-ALK*^[21]^. ALK mutations are often found in never or light smokers and younger populations. Common ALK inhibitors include alectinib, brigatinib, certinib, lorlatinib and crizotinib. In the ALEX study, 303 patients were randomly selected to receive alectinib versus crizotinib. Alectinib showed a decreased risk of progression or death in 53% of patients, PFS was 35 months versus 11 months in the crizotinib arm and OS was not reached^[22]^. Common side effects included anemia, myalgia, hyperbilirubinemia, weight gain and photosensitivity.

### MET mutations

MET is a tyrosine kinase receptor for hepatocyte growth factors. MET mutations include MET exon-14 skipping mutations (3% of NSCLC) and *MET* gene amplification (2%-4% of NSCLC). MET exon-14 skipping mutations decreases the degradation of MET which causes it to become a oncogenic driver. Capmatinib, crizotinib and tepotinib have been both approved for treatment of MET exon-14 skipping mutations. In the GEOMETRY-mono-1 trial included 97 patients with MET exon-14 skipping mutations and capmatinib showed a 68 percent overall response rate and the PFS was 12.4 months. Common adverse reactions included peripheral edema, nausea, vomiting and increased creatinine^[23]^. Lastly, in *MET* gene amplifications capmatinib or crizotinib is often used.

### RET rearrangements

The rearranged during transfection gene (*RET*) translates a cell surface tyrosine kinase receptor kinase that is mutated. RET rearrangements are present in 1%-2% of NSCLC, often in non-smokers and younger patients. First line therapy include treatment with selpercatinib, pralsetinib and cabozantinib. Common side effects include hypertension, fatigue, diarrhea, transaminitis and pneumonia^[24-26]^.

### BRAF mutations

BRAF is involved with downstream signaling of Kristen rat sarcoma viral oncogene homolog (KRAS) and activates the mitogen-activated protein kinase (MAPK) pathway. BRAF mutations are present in 1%-3% of NSCLC and are present in patients with a smoking history. BRAF mutations can occur in the V600 position of exon 15 or outside of the domain. First line therapy options include dabrafenib, dabrafenib/trametinib and vemurafenib.

### NTRK fusions

NTRK fusions are very rare (< 1% of NSCLC) and involve one of three tropomyosin receptor kinases (TRK). Therapy options for NTRK fusions include Larotrectinib and Entrectinib. Larotrectinib was analyzed in several phase I/II trials which showed an ORR 79%. Most common adverse effects included elevated transaminases, anemia and neutropenia^[27]^. Entrectinib showed a ORR 57% in several trials^[28]^. Entrectinib and Larotrectinib have not been compared with each other.

### KRAS mutations

The Kristen rat sarcoma viral oncogene homolog (KRAS) occurs in 20% to 25% of NSCLC and is often associated with smoking^[29]^. In NSCLC, the G12C mutation is targeted by sotorasib and adagrasib as subsequent therapy after patients have received 1 prior therapy. The most common adverse reactions include elevated transaminases and diarrhea.

### Mechanisms of resistance

**Figure 1:**
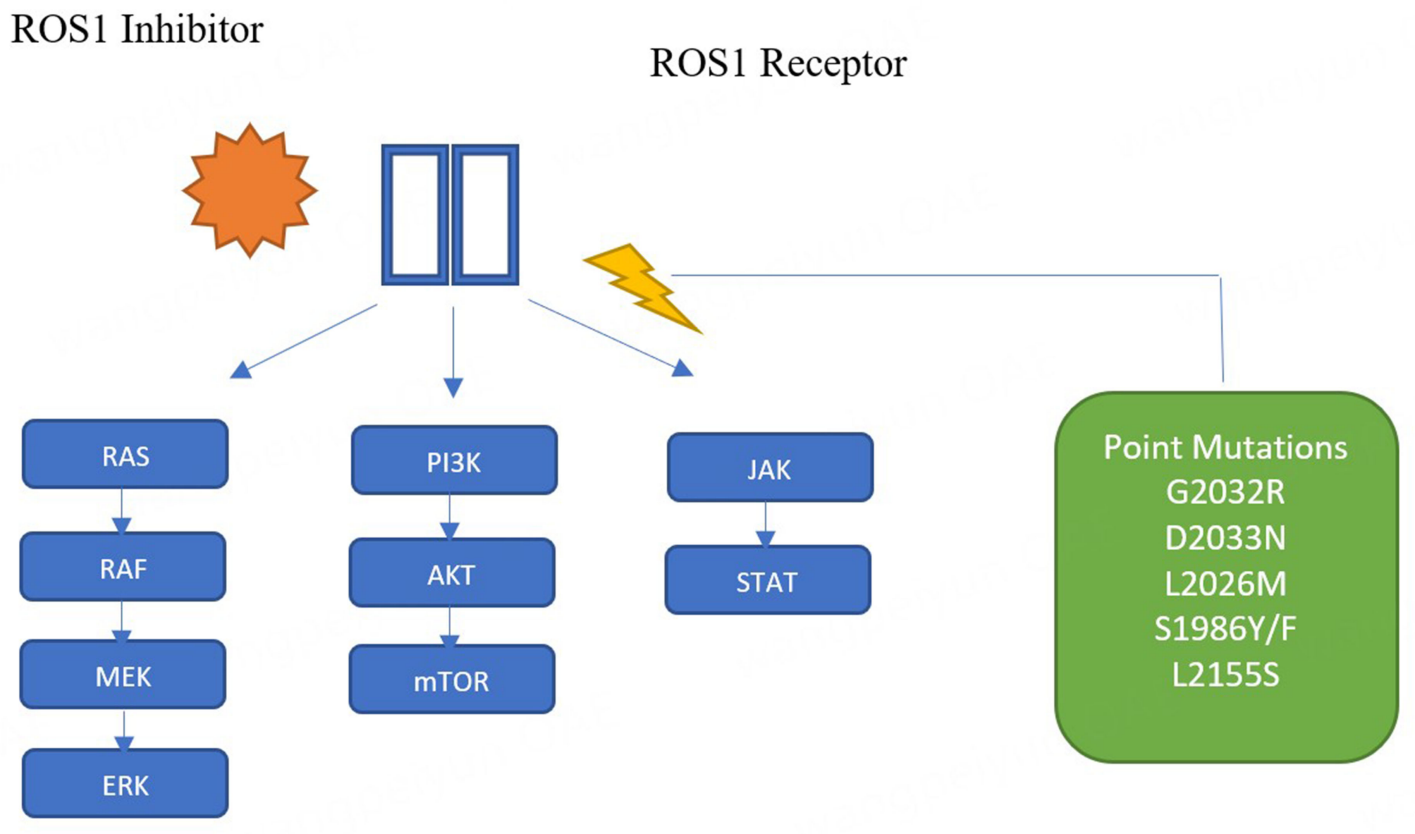
ROS1 pathway and resistance mechanism.Molecular mechanisms of ROS1 Inhibitors in ROS-1 rearranged lung cancer patients. Molecular pathways to resistance include an activated ROS1 kinase which activates the SHP-2 phosphatase and increases the JAK/STAT3, PI3K/AKT/mTOR, RAS/RA/MEK/ERK signaling pathways to promote growth and survival of the cell.

As was mentioned before, systemic or central nervous system resistance often develops after good clinical responses and survival. Mechanisms of resistance to ROS1 can be categorized as on-target or off-target. After a tumor has been treated with a TKI, on-target resistance mechanisms can emerge, such as ROS1 mutations^[30-32]^. There are several mutations described, the most commonly recognized of which is G2032R, followed by D2033N, and both of them are solvent-front mutations. Other acquired resistance mutations included gate keeper mutations and among them S1986F/Y, L2000V, F2004V, L2026M, and G2032K^[33-36]^. L2086F is an important and very dangerous resistance mutation because it is resistant to all TKIs (crizotinib, lorlatinib, taletrectinib) [Figure 1]^[30,31,34]^.

TKIs have varying efficacy against resistant mutations, sensitivity must be confirmed prior to initiating therapy. For example, lorlatinib has efficacy against the K1991E or S1986F resistant mutations, but has limited therapeutic potential against the G2032R mutation^[35,36]^, and may be used after failure on entrectinib^[37]^. Sequential use of crizotinib and lorlatinib has led to the formation of a compound mutation of G2032R/L2086F but cabozantinib has potential to overcome this compound mutation^[30,31,34]^. Cabozantinib is frequently used to treat thyroid cancer as well as other tumors that it can selectively target, including MET, VEGFR-2, RET, ROS-1, and AXL, with good penetration of blood brain barrier. Despite the presence of resistance mutations such as D2033N or G2032R^[38-40]^, resistance to crizotinib, ceritinib, and entrectinib can be overcome with cabozantinib. Brigatinib, an additional ROS1 inhibitor, has demonstrated antitumor activity against a number of crizotinib-resistance mechanisms^[41]^, including the L2026M mutation, but not the G2032R mutation^[41-43]^. Taletrectinib, ROS1 and NTRK inhibitor with activity against G2032R, L1951R, S1986F and L2026M mutations, but minimal activity against D2033N mutation. A clinical trial with 46 patients on previous multiple lines of therapy had a 33% ORR and another trial with 15 patients, reported 58.3% ORR in previously exposed patients and 66.7% in treatment naïve patients^[30,44]^. There have been additional intrinsic mutations discovered in addition to the ones described and shown in Table 1.

**Table 1:** Intrinsic mutations in ROS1 resulting in resistance

**Intrinsic**	**Crizo** **tinib**	**Ceri** **tinib**	**Lorla** **tinib**	**Briga** **tinib**	**Cabozan** **tinib**	**Fore** **tinib**	**Entrec** **tinib**	**Repotrec** **tinib**	**Ensar** **tinib**
G2032 R/K	R	R	R	R	S	S	R	S	R
D2033	R	R	S	R	S	S	R	S	R
L1951	R	R		R	S				
L2026	R	R	S	R	S	S	R		
L1196M	R								
S1986 Y/F	R	R	S						
L2086F	R								
E1935G	R	R				S			
L1947R	R					S			
G1971E	R					S			
L1982F	R				S	S			
C2060G	R					S			
V2098I	R	S		S	S	S			
L2155S	R	R							
E1990G	R	R			S				
F1994L	R	R			S				
F2004 C/I	R	R	S	S	R	S			

Blank: No available data; R: resistant; S: sensitive.

Additionally, there are important off-target resistance mechanisms that involve the presence of other genomic aberrations like: *MET* amplification (3%), *KRAS* mutations (20%-25%) or small cell transformation (3%-10%)^[45-47]^. Lastly, the molecular pathways to resistance includes an activated ROS1 kinase activates the SHP-2 phosphatase and increases the JAK/STAT3, PI3K/AKT/mTOR, RAS and MAP/ERK signaling pathways to promote growth and survival of the cell^[48]^ and HER2 mediated bypass signaling [Figure 1].

## ROS1 TARGETED THERAPIES

Numerous tyrosine kinase inhibitors (TKIs) have been developed, such as crizotinib, ceritinib, entrectinib, and lorlatinib. Both crizotinib and entrectinib have been approved for use in the first-line setting. According to Table 2, the use of a ROS1 TKI in the first-line setting improves OS, response rate (RR), and progression-free survival (PFS) in comparison to standard chemotherapy^[24]^. Table 3 depicts the most prevalent adverse events associated with each targeted therapy.

**Table 2:** Clinical activity and toxicity profiles of ROS1 tyrosine kinase inhibitors in treatment naïve patients

**TKI**	**RR (%)**	**PFS (months)**	**OS (months)/(1 yr)**
Crizotinib^[49,50]^	72	19.3	51.4/79
Ceritinib^[56]^	67	19.3	24/56
Entrectinib^[62]^	53	19	NR/85
Lorlatinib^[64]^	21	21	NR

OS: Overall survival; PFS: progressive free survival; RR: response rate; TKI: Tyrosine Kinase Inhibitor.

**Table 3:** Summary of adverse events from ROS1 tyrosine kinase inhibitors

**Tyrosine Kinase Inhibitor**	**Common TRAE**	**Grade 3-4 TRAE (%)**
Crizotinib^[49,50]^	Visual impairment, diarrhea, constipation, peripheral edema, nausea, elevated AST, dizziness	36
Ceritinib^[56]^	Diarrhea, nausea, anorexia, vomiting, cough, elevated creatinine, elevated transaminases	37
Entrectinib^[62]^	Dysgeusia, fatigue, dizziness, constipation, nausea, weight gain, paresthesia	34
Lorlatinib^[64]^	Hypercholesterolemia, hypertriglycerdiemia, edema, peripheral neuropathy, AMS, weight gain, dizziness	49

AMS: Altered mental status; AST: aspartate aminotransferase; TRAE: treatment related adverse events.

### Crizotinib

Crizotinib was the first TKI to be approved for management of ROS1 mutated NSCLC. In 2014, the PROFILE 1001 study showed significant activity against ROS1-rearranged NSCLC^[49]^. PROFILE 1001 was a multicenter, single-arm phase I study that included patients with metastatic ROS1 positive NSCLC that depicted an ORR 72%, PFS 9.2 months and median DOR 17.6 months^[49]^. An updated analysis of the PROFILE 1001 in 2019, median OS was 51.4 months, median PFS 19.3 months and median DOR 24.7 months^[50]^.

In a prospective phase II OxOnc study, crizotinib demonstrated an ORR 91%, DOR 19.7 months, PFS 15.9 months and OS 32.5 months^[51]^. In comparison to the PROFILE 1001 study, the OxOnc study included patients with brain metastases (BM) both symptomatic and asymptomatic comprising 18% of study population. The study showed a PFS of 10.2 months (patients with BM) *vs.* 18.8 months (patients without BM)^[51]^. Additionally, another phase II multicenter study, evaluated not only ROS1 rearrangement but also co-mutations with TP53. The results of the phase II study showed ORR 70% with median PFS 20 months, while those with TP53 co-mutations had a shorter median of PFS of 7 months^[52]^.

Failure to respond to crizotinib frequently results from CNS progression and mutations. G2032R/K, D2033N, S1986Y/F, L2026M, and L1951R are common mutations that cause crizotinib to fail, with G2032R being the most common^[53]^. Lorlatinib is a potential agent that can be used in patients that have progression on crizotinib as it is able to overcome resistance mutations.

In a phase I/II trial with ROS1-positive patients who have received crizotinib previously led to an ORR of 35%^[54]^. Additionally, cabozantinib is a potential agent that be used to overcome crizotinib resistance^[38]^. Crizotinib also has very poor blood-brain barrier penetration and is often limited for use in patients with CNS progression^[53]^. Lastly, common side effects of crizotinib include vision disorder, diarrhea, nausea, vomiting and peripheral edema^[49]^.

### Ceritinib

Ceritinib is a second-generation TKI approved to treat NSCLC patients with ROS1 rearrangement. Ceritinib is a highly potent TKI with a potency 20 greater than crizotinib^[55]^. Ceritinib efficacy was evaluated in a multicenter, phase II study of 32 patients with ROS1 rearrangement and the results showed a ORR of 67%, median PFS 19.3 months and median OS 24 months^[56]^. Most common side effects included diarrhea, nausea and anorexia^[56]^. In 2017, the ASCEND-8 trial evaluated if a decreased dosage of ceritinib had similar efficacy with less adverse risk. Certinib 450 mg was compared to the standard 750 mg dosage and similar efficacy but more tolerable side effects were seen using the lower dosage^[57]^. Although ceritinib is a highly effective TKI in the treatment of ROS1-mutated NSCLC, it has limited applications in crizotinib-resistant patients because it is resistant to the common mutations seen with crizotinib, such as G2032R, D2033N, L1951R, and S1986Y/F^[55]^.

### Entrectinib

Entrectinib is a ROS1 inhibitor with profound penetration in the CNS allowing it to exert its anti-tumor activity^[58]^. In the STARTRK-1, STARTRK-2 and ALKA-372-001 trials, 53 ROS1 mutated treatment naïve patients were given entrectinib 600 mg daily and followed for at least 12 months. The patients were evaluated for ORR and DOR as co-primary endpoints while OS, PFS, intracranial DOR (IC-DOR), intracranial ORR (IC-ORR) and safety effect profile were secondary endpoints. In the studies, most patients were white (59%), female (64%) and never smokers (59%) with baseline CNS disease present in over 43% of patients^[59-61]^. Forty-one patients were found to have a response, 6% of patients had a complete response (CR), 72% had a partial response (PR) and 2% had an objective response (OR). The median DOR was 24.6 months, ORR 77% and median PFS 19 months in patients without CNS disease. Median OS was not met at 15.5 months during follow-up. While on the contrary, patients with CNS disease had a median DOR 12.6 months, ORR 74%, median PFS 13.6 months, IC-DOR 12.9 months and IC-ORR was 55%. The study showed that entrectinib was not only active systemically but also had good CNS penetration. The most common side effects included nervous system disorders (3%) and cardiac disorders (2%)^[62]^.

### Lorlatinib

Lorlatinib is a selective third-generation ALK and ROS1 TKI that has both systemic and CNS activity via reduction of P-glycoprotein 1-mediated efflux^[63]^. In a phase I study, Lorlatinib was given to ROS1-positive NSCLC patients with ECOG status 0-1. The primary endpoint was dose-limiting toxicities and the secondary endpoints were pharmacokinetics, safety, and overall response. Lorlatinib has good penetration in the CNS with a response of 60%. Common adverse effects were weight gain, peripheral edema and constipation.

In a phase II trial, 69 patients were enrolled with ROS-1 positive NSCLC with 30% being TKI naïve and 70% having previous TKI exposure (58% were pretreated with crizotinib and 12% with other TKIs) to evaluate for an overall and intracranial response. The RR was 62% in the TKI naïve patients and 35% in the TKI exposed patients, while the PFS was 21 months *vs.* 8.5 months. Intracranial responses were seen in 64% of TKI naïve and 50% in those pre-treated with crizotinib. The most common side effects included hypertriglyceridemia and hypercholesteremia^[64]^.

In the PFROST study, predictive molecular events for response were evaluated. All patients in study were ROS1 mutated and had been previously treated with crizotinib. Prior to initiation of therapy with lorlatinib, the patients had a tissue or blood sample taken. Patients with a G2032R mutation progressed rapidly and continued to have this mutation at the time of treatment failure, showing the importance of early testing to predict response to therapy^[35]^.

Lastly, the efficacy of lorlatinib was tested in the French LORLATU and Asian GLASS studies, which revealed significant RR both extracranial and intracranial, confirming lorlatinib’s potency as a good therapeutic option in the treatment of ROS1 mutated NSCLC^[65,66]^.

### Novel agents

First-generation TKIs are effective in treating ROS1+ NSCLC until resistance mutations develop, presenting a clinical challenge. Chemotherapy is still used as a last resort after disease progression occurs as a result of resistance mutations. Chemotherapy is not as effective as TKIs, with a median PFS of 7 months^[44]^. Studies for potential newer second-line regimens are ongoing.

### Cabozantinib

Cabozantinib is a tyrosine kinase inhibitor that is currently approved for treatment of renal cell carcinoma, hepatocellular carcinoma and medullary thyroid cancer^[39]^. In a recent study, four patients were evaluated that developed resistance to first line TKIs, crizotinib and ceritinib, and were subsequently treated with cabozantinib. In the study, an OR 25 percent and PFS from 4.9 to 13.8 months was achieved. Study limitations included no pre-therapy tissue samples to identify resistance mutations and no clear understanding whether the effects were due to ROS1 inhibition or direct drug effects^[67]^. Cabozantinib is very effective against most mutations including G2032R and D2033N but has significant toxicity that limits research on the medication for ROS1+ patients^[58]^.

### Taletrectinib (DS-6051b)

Taletrectinib is a TKI that has activity against both NTRK and ROS1. Recently, it was found that Taletrectinib can be used in ROS1 positive patients that harbor CD74, S1986F, L2026M, L1951R and G2032R mutations^[39]^. In a phase I study, 15 Japanese patients were enrolled with 12 having measurable lesions and 9 were treatment naïve. In treatment naïve patients, the ORR was 66.7 percent, while those that were pre-treated with TKIs the ORR was 33.3 percent^[44]^. Most recently, two phase I studies in the United States and Japan evaluated an ORR, PFS and safety. Twenty-two patients with ROS1 positive NSCLC were evaluated and given Taletrectinib in dose escalation. The ORR for ROS1 TKI naïve patients was 66.7 percent, while for previously treated patients it was 33.3 percent. The PFS for TKI naïve patients was 29.1 months, in contrary to pre-treated TKI patients it was 14.2 months^[68]^. Taletrectinib was found to have good systemic activity in patients with ROS1 positive NSCLC regardless of TKI pretreatment. Most common side effects include elevated transaminases (27%) and GI toxicity (4.5%).

### Repotrectinib (TPX-0005)

Repotrectinib is a selective tyrosine kinase inhibitor that not only inhibits NTRK and ALK but also ROS1 with a potency level of > 90 folds of crizotinib and is able to overcome G2032R and D2033N mutations^[69]^. Repotrectinib has both systemic and CNS activity^[70]^. Recently, in the TRIDENT-1 trial, 33 ROS1 positive patients were evaluated that included both TKI naïve and pre-treated TKI patients. The patients received dose escalations of repotrectinib with a ORR 82 percent (regardless of dose) in TKI naïve patients and 39 percent in pre-treated TKI patients. The ORR increased to 55 percent in pre-treated TKI patients with dosages of 160 mg. The intracranial-ORR was 100 percent in treatment naïve patients and 75 percent in those previously treated with TKI. The median DOR was 23.1 months, while the PFS was 24.6 months^[71]^. The study demonstrates that repotrectinib is a potent TKI that can be used in both treatment naive and previously treated TKI patients with ROS1 positivity. More research is required, and it is currently underway in phase II of the Trident study, which is scheduled to conclude in 2023. The TRIDENT study’s Phase II is enrolling 190 ROS1 positive patients and evaluating response based on the number of TKIs previously used. Dizziness, fatigue, constipation, and dyspnea are the most common side effects.

## CONCLUSIONS

ROS1 fusions are not very common and only represent around 2% of NSCLC, however due to the high incidence of lung cancer in the US, this genetic aberration is present in several thousand patients.

Thanks to DNA NGS and other diagnostic methods in tissue or blood we are able to detect these genetic aberrations and offer our patient’s front-line therapy with very good clinical outcomes, significant survival prolongation and good quality of life.

Currently, crizotinib and entrectinib are approved for ROS1 tumors, however ceritinib and lolartinib have already showed good clinical efficacy too. Repotrectinib, talotrectinib and cabzantinib are in development to fight on-target mechanisms of resistance as future therapeutics in the combat against ROS1 fusions.

## DECLARATIONS

### Copyright

© The Author(s) 2023.
